# Altered cerebellar lobular volumes correlate with clinical deficits in siblings and children with ASD: evidence from toddlers

**DOI:** 10.1186/s12967-023-04090-x

**Published:** 2023-04-07

**Authors:** Manoj Kumar, Chandrakanta Hiremath, Sunil Kumar Khokhar, Eshita Bansal, Kommu John Vijay Sagar, Hansashree Padmanabha, Akhila S. Girimaji, Shweta Narayan, M. Thomas Kishore, B. K. Yamini, A. R. Jac Fredo, Jitender Saini, Rose Dawn Bharath

**Affiliations:** 1 Department of Neuroimaging and Interventional Radiology, National Institute of Mental Health and Neurosciences (NIMHANS), Hosur Road, Bengaluru, 560029 India; 2 Department of Child and Adolescent Psychiatry, National Institute of Mental Health and Neurosciences (NIMHANS), Hosur Road, Bengaluru, India; 3 Department of Neurology, National Institute of Mental Health and Neurosciences (NIMHANS), Hosur Road, Bengaluru, India; 4 Department of Speech Pathology and Audiology, National Institute of Mental Health and Neurosciences (NIMHANS), Hosur Road, Bengaluru, India; 5 Department of Clinical Psychology, National Institute of Mental Health and Neurosciences (NIMHANS), Hosur Road, Bengaluru, India; 6 School of Biomedical Engineering, Indian Institute of Technology (BHU), Varanasi, Uttar Pradesh, India

**Keywords:** Autism spectrum disorder, Behavioral and developmental assessment, Cerebellum, Lobule, Magnetic resonance imaging, Volumetric analysis

## Abstract

**Background:**

Autism Spectrum Disorder (ASD) is a neurodevelopmental disorder characterized by impaired social and communication skills, narrow interests, and repetitive behavior. It is known that the cerebellum plays a vital role in controlling movement and gait posture. However, recently, researchers have reported that the cerebellum may also be responsible for other functions, such as social cognition, reward, anxiety, language, and executive functions.

**Methods:**

In this study, we ascertained volumetric differences from cerebellar lobular analysis from children with ASD, ASD siblings, and typically developing healthy controls. In this cross-sectional study, a total of 30 children were recruited, including children with ASD (N = 15; mean age = 27.67 ± 5.1 months), ASD siblings (N = 6; mean age = 17.5 ± 3.79 months), and typically developing children (N = 9; mean age = 17.67 ± 3.21 months). All the MRI data was acquired under natural sleep without using any sedative medication. We performed a correlation analysis with volumetric data and developmental and behavioral measures obtained from these children. Two-way ANOVA and Pearson correlation was performed for statistical data analysis.

**Results:**

We observed intriguing findings from this study, including significantly increased gray matter lobular volumes in multiple cerebellar regions including; vermis, left and right lobule I–V, right CrusII, and right VIIb and VIIIb, respectively, in children with ASD, compared to typically developing healthy controls and ASD siblings. Multiple cerebellar lobular volumes were also significantly correlated with social quotient, cognition, language, and motor scores with children with ASD, ASD siblings, and healthy controls, respectively.

**Conclusions:**

This research finding helps us understand the neurobiology of ASD and ASD-siblings, and critically advances current knowledge about the cerebellar role in ASD. However, results need to be replicated for a larger cohort from longitudinal research study in future.

## Introduction

Autism Spectrum Disorder (ASD) is a pervasive neurodevelopmental disorder with multifactorial etiology for poorly understood brain mechanisms. Poor social cognition and reciprocity, communication deficits, and restricted and repetitive behaviors are core characteristics of ASD symptoms [1]. It has also been well reported that the cerebellum was crucial in controlling movement and gait posture. Due to complexity and late-maturing, cerebellar neural regions are vulnerable to early developmental insults that can profoundly disturb intracerebellar development [2, 3]. The cerebellum has a protracted growth trajectory and develops throughout pregnancy; however, rapid growth is observed in the third trimester and may continue in the first postnatal year [4–6]. Due to the protracted development, the cerebellar circuitry is very vulnerable in the early days and weeks following birth [6], a period during which the cellular makeup and the quantity of inputs change quickly [7], and cortical areas continue to mature for more years [8]. Consequently, cerebellar developmental disturbances are believed to impair proper cerebellar-cortical circuit formation, the most robust finding in ASD. Initially, it was documented that the cerebellum is conventionally associated with motor functions; however, through recent studies, it has been well established that apart from the motor function, the cerebellum is also an essential structure within the social circuitry, visuospatial, language, and cognitive functional domains [9]. Cerebellar neuroanatomical alterations are among the most replicated findings in post-mortem brain samples of patients with ASD [10]. Willsey et al. has reported the co-expression of ASD-related genes during early brain development in the cerebellum, which is implicated in the pathogenesis of ASD [11]. Thus, the cerebellum grows during a known genetic and environmental vulnerability period and reaches full size to guide the refinement of neocortical structures [12].

Cerebellum has been a well-documented brain structure and considers an “*old brain*” with a unique topographic organization [14, 15]. Each region is attributed with a separate function based on its specific spatiotemporal location and connectivity associated with other brain regions related to multiple functions, including motor, coordination visuospatial, learning, and balance. Along with the above-mentioned functional features, recent studies have demonstrated that the cerebellum also plays a pivotal role in social cognition and executive functions [13, 15]. The cerebellum is broadly divided into three lobes and ten lobules, and these lobes can be classified as the anterior lobe (lobule I–V), posterior lobe (lobule VI–IX), and the flocculonodular lobe (lobule X) based on their neuroanatomical locations. Previous literature from neuroimaging on differentiating between individuals with ASD and healthy controls posits a distinction in various cerebellar regions. Several researchers using structural imaging have described the arrested growth of the posterior vermis [16, 17]. The consistent findings from neuroimaging studies reported on the reduction of gray matter in right Crus I, lobule VIII, and lobule IX using voxel-based morphometry [19, 20]. In recent research, several studies reported a correlation between regional cerebral volumes and sensorimotor abilities in the ASD population [21, 22]. However, only a few studies have been conducted to assess the covariation of cerebellar morphometry at the lobular level and socio-behavioral and motor functions in ASD in a very young children population.

Functional MRI studies have also suggested decreased activity in the cerebellum in the ASD group during social, language, and motor tasks [22]. It has also been well reported that children with ASD failed to engage the anterior cerebellum (lobule IV/V) while performing motor tasks compared to healthy controls. Using structural MRI in an adolescent population of ASD, D’Mello et al. have reported a decrease in gray matter volume in ASD children in cerebellar lobule VII (Crus I/II) compared to healthy controls [23]. This reduction in regional and lobular gray matter volume in distinct cerebellar subregions was correlated with the severity of social interaction, communication, and repetitive behaviors in children with ASD [23].

Therefore, the primary aim of this study was to examine volumetric lobular measurements in toddlers with ASD, ASD siblings, and typically developing children; also, to explore the correlation between lobular volume and clinical (developmental and behavioral) measures between these groups. To the best of our knowledge, this is the first attempt to know whether there is a volumetric difference at lobular levels in 34 cerebellar lobules between three groups at an early age (i.e., in toddlers). The findings reported in this study will provide knowledge to the existing literature in understanding the role of the cerebellum in the etiopathophysiology of ASD.

## Material and methods

### Study participants

This prospective cross-sectional study was performed on thirty children aged between 15 and 36 months who participated in this study. Out of a total of 30 study participants, ASD children (N = 15; mean age = 27.67 ± 5.1 months), ASD siblings (N = 6; mean age = 17.5 ± 3.79 months), and typically developing children (N = 9; mean age = 17.67 ± 3.21 months) were included in this study. All the subjects (ASD, ASD siblings, and typically developing children) who participated in this study were recruited from a tertiary care hospital setting. An institutional ethics committee approved the study, and written informed consent and assent were obtained from the parent(s) or guardian(s) of the children recruited in this study.

The clinical diagnosis for ASD children was confirmed by experienced child psychiatrists clinically based on the DSM-V criteria. Further ASD diagnosis was also confirmed by the AIIMS IN-CLEN diagnostic tool [24] for children aged 2 and above 2 years of age. However, the Modified Checklist for Autism in Toddlers-Revised (M-CHAT-R) screening tool [25] was used for children ages 16 months and 2 years of age to classify children at high risk for ASD.

### Inclusion and exclusion criteria for ASD

Children aged between 15 and 36 months and considered to be at risk for ASD based on early developmental/behavioral assessment and modified checklist for autism in toddlers-revised (M-CHAT-R) screening tools [25].

The following inclusion and exclusion criterion were used for recruitment. Children between 15 and 36 months considered at risk for ASD based on early developmental/behavioral assessment by M-CHAT-R at 16 months were included. Children with known neurological injury or disorders such as cerebral ischemia, inflammation, infection, perinatal, postnatal trauma, chromosomal/congenital anomalies affecting CNS development, or toddlers with global developmental delay without autism were excluded.

### Inclusion and exclusion criteria for typically developing children

Typically developing children aged between 15 and 36 months without any known neurological and neurodevelopmental conditions were included in this study. Control participants were screened using the Developmental screening test (DST) to assess their development in various domains such as motor, language, social and congnition. The following exclusion criteria were applied for the healthy control group, including diagnosed, referred, or suspected condition of ASD or any other neurodevelopmental disability. Typically developing children have a first or second-degree relative with ASD were also excluded from the study.

If the typically developing children met the inclusion and exclusion criteria mentioned above, then further neurodevelopmental assessment, namely the Vineland Social Maturity Scale (VSMS) [26] and Bayley Scales of Infant and Toddler Development (Bayley-III) [27], were administered by a trained clinical psychologist, to note the degree of adaptive behavior and development in cognitive, language, and motor domains, respectively.

### MRI data acquisition

Conventional and advanced MRI scanning was performed with identical acquisition parameters on all the participating subjects on a 3.0 T clinical MRI (Skyra, Siemens, Erlangen, Germany) using a 32-channel transmit received head coil. All MRI scans were completed under natural sleep (without any sedation). We used a simple approach of applying silicone earplugs for noise protection, swaddling, and feeding at least 30 min before the MRI scan to facilitate faster and more deep natural sleep. Each participant's parent was present throughout the MRI scan inside the MRI room to consolidate the child and ensure the child was comfortable. The vital physiological parameter monitoring, including arterial oxygen saturation and heart rate, were continuously performed throughout the MRI examination in every subject. A continuous monitoring for signs of awakening and head movements were done using a camera throughout the MRI scan, which was extremely valuable in ensuring quality data collection. High-resolution axial T1-weighted images were acquired on each participating subject using the following imaging acquisition parameters, including a field of view (FOV) = 220 × 220 mm; repetition time (TR) = 8.5 ms; echo time (TE) = 3 ms; inversion time (TI) = 1610 ms, flip angle = 13°, voxel resolution = 1 × 1 × 1mm^3^; respectively.

### MR image processing and data analysis

After successfully completion of MRI scan, the raw DICOM MRI data were transferred to an offline workstation dedicated to image processing and quantitative analysis. The raw DICOM images from individual subjects were converted to NifTI file format using a standard dicom2nii conversion script, and image preprocessing and quality assurance pipelines were performed. During the preprocessing and data quality checks steps, MRI data from five participanting subjects were excluded from the study due to motion and distorted images that precluded the series from being used (ASD, n = 3; ASD-sibling, n = 1; Healthy control = 1). Within each group, the excluded participants did not differ from included participants for age, IQ, or other clinical and behavioral features from their peer’s group.

### Lobular volume analysis

The cerebellum was preprocessed individually by using the Spatially Unbiased Infratentorial Template (SUIT) toolbox [28] implemented in the statistical parametric mapping (SPM-8; Wellcome Department of Imaging Neuroscience, UK), and cerebellar lobular volumes were extracted using ITK-SNAP tools [29]. In summary, we have used the following data processing and image analysis pipeline to extract and measure the cerebellar lobular volume in this study.

**Fig. 1:**
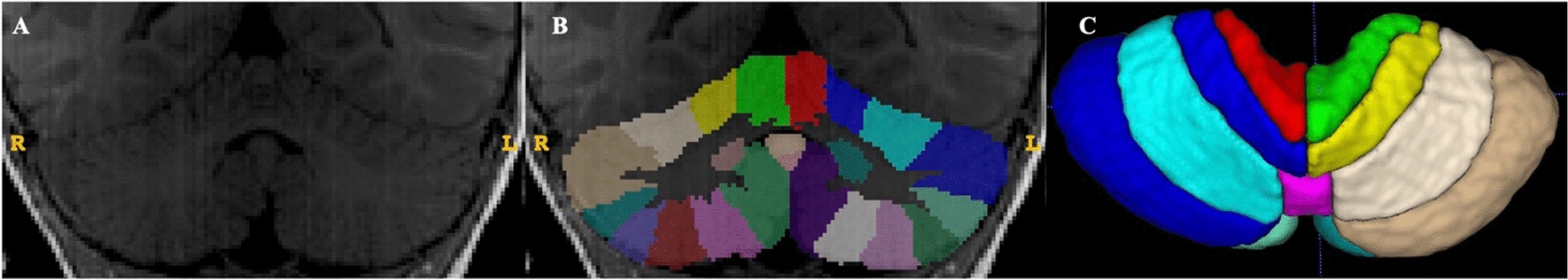
A representative T1-weighted coronal image demonstrating the cropped cerebellar structure from the cerebrum (**A**). **B** reveals the segmented cerebellar lobules overlaid on the cropped cerebellum. **C** shows the 3D mesh from the segmented cerebellar lobules

- **Preprocessing:** before starting the preprocessing steps, all the DICOM data was converted into NIFTI file format. AC-PC corrections were performed for data alignment and AC-PC line of correction.
- **Cropping and isolation:** AC-PC corrected T1 weighted anatomical images were used to perform the cerebellar cropping and isolation of the cerebellum from the cerebrum. Subsequently, a manual cerebellar mask correction was performed by overlaying the original isolated cerebellar T1W image with a segmented cerebellar mask image using ITK-SNAP tool. The individual cerebellar mask was manually checked and corrected for any mismatch between cropped cerebellar image and overlaid cerebellar mask.
- **Normalization:** In the following steps, the isolated cerebellum was normalized into SUIT template space. An inverse transformation was applied to convert the cerebellar lobular atlas into the probabilistic cerebellar atlas into individual subject space using the deformation parameters from the normalization.
- The modulated gray matter probability maps were also smoothed using an 8-mm FWHM Gaussian kernel. Additionally, every participant's modified driven equilibrium fourier transform (MDEFT) was segmented in SPM to estimate the total gray matter volume. The whole cerebellar volume was also measured to perform the normalization of cerebellar lobular volume to correct for global brain size-induced variations from individual subject data (Fig. 1).

### Statistical analysis

Continuous variables were expressed as mean ± SD and categoric variables as frequencies and percentages. Demographic and developmental assessment data were tested for normality using the Kolmogorov–Smirnov test. Two-way ANOVA was done since there were three groups, including children with ASD, ASD-sibling, and typically developing healthy controls, to examine the difference in the volume of cerebellum lobules across the three groups. The correlation between cerebellum lobule volume and developmental measures such as adaptive behaviors (as indicated by the social quotient on VSMS), cognition, language, and motor development (as indicated by developmental quotients of BSID-III) were calculated using Pearson correlation. Cerebellar lobular volumes were corrected with total cerebellar volume (individual cerebellar lobular volumes/total cerebellar volume). Results were considered significant at p-values < 0.05 after FWE cluster-level correction (clusters formed with p < 0.005 at the corrected level). All the statistical computations were carried out using Statistical Package for Social Sciences (SPSS, version 22.0, Armonk, NY: IBM Corp. and R-Stats Package software version 3.5.0, Boston, MA, USA). A p-value of ≥ 0.05 was statistically significant.

## Results

### Subjects characteristics

#### Neurodevelopmental and cognitive developmental scores

**Fig. 2:**
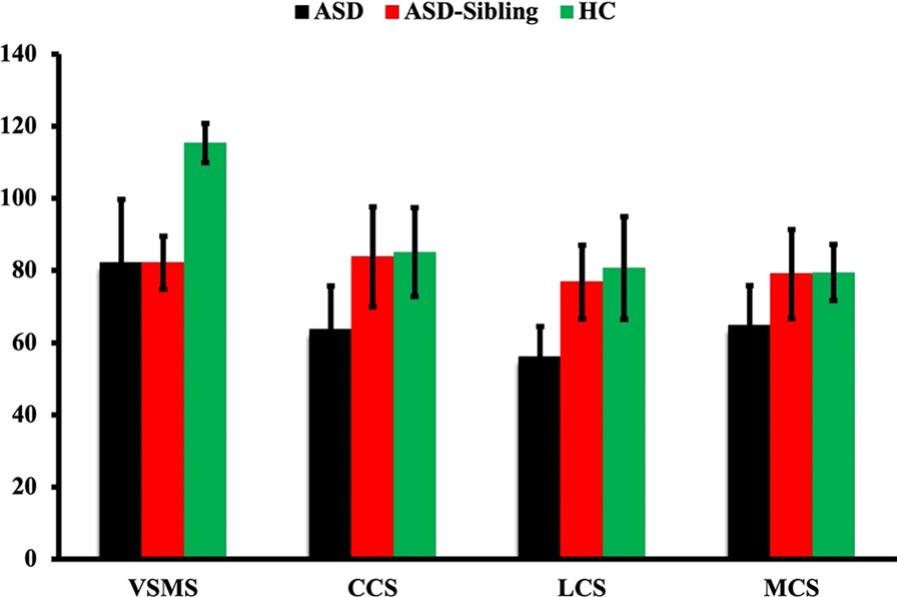
Bar graph representing the neurodevelopmental assessment derived social quiescent scores (VSMS) and composite cognitive, language, and motor scores derived from Bayley-III assessment from ASD, ASD siblings, and healthy controls. *VSMS* Vineland social maturity scale, *CCS* cognitive composite score, *LCS* language composite score, and *MCS* motor composite score, respectively

Our neurodevelopmental data derived from the VSMS, and Bayley-III scales demonstrate significant differences between ASD and healthy controls in all the assessed domains, i.e., quiescent social scores, social cognition, language, and motor functions (Table 1 and Fig. 2).

**Table 1:** Demographic and Clinical characteristics and Neurodevelopmental assessments from study participants

Demographic Measures			
Demographic Details	**ASD**	**ASD Sibling**	**Healthy Control**
No. of participants	N = 12	N = 5	N = 8
Mean Age (Months)	27.6 ± 5.05	17.5 ± 3.79	17.67 ± 3.21
Gender: Male (Female)	9 (3)	1 (3)	8 (2)
Clinical and Developmental Measures			
M-CHAT-R	11.25	1	0
VSMS Scores	82.05 ± 17.62	82.06 ± 7.32	115.29 ± 5.40
BAYLEY-III			
Composite Cognitive Scores	63.75 ± 11.89	83.75 ± 13.77	85 ± 13.33
Composite Language Scores	56.08 ± 8.43	76.75 ± 10.21	80.67 ± 14.22
Composite Motor Scores	64.75 ± 10.94	79 ± 12.25	79.33 ± 7.77

*BAYLEY-III* Bayley scales of infant and toddler development, 3rd edition, *M-CHAT-R* Modified checklist for autism in toddlers-revised, *VSMS* Vineland social maturity scale

### Cerebellar lobular volumetric changes

**Fig. 3:**
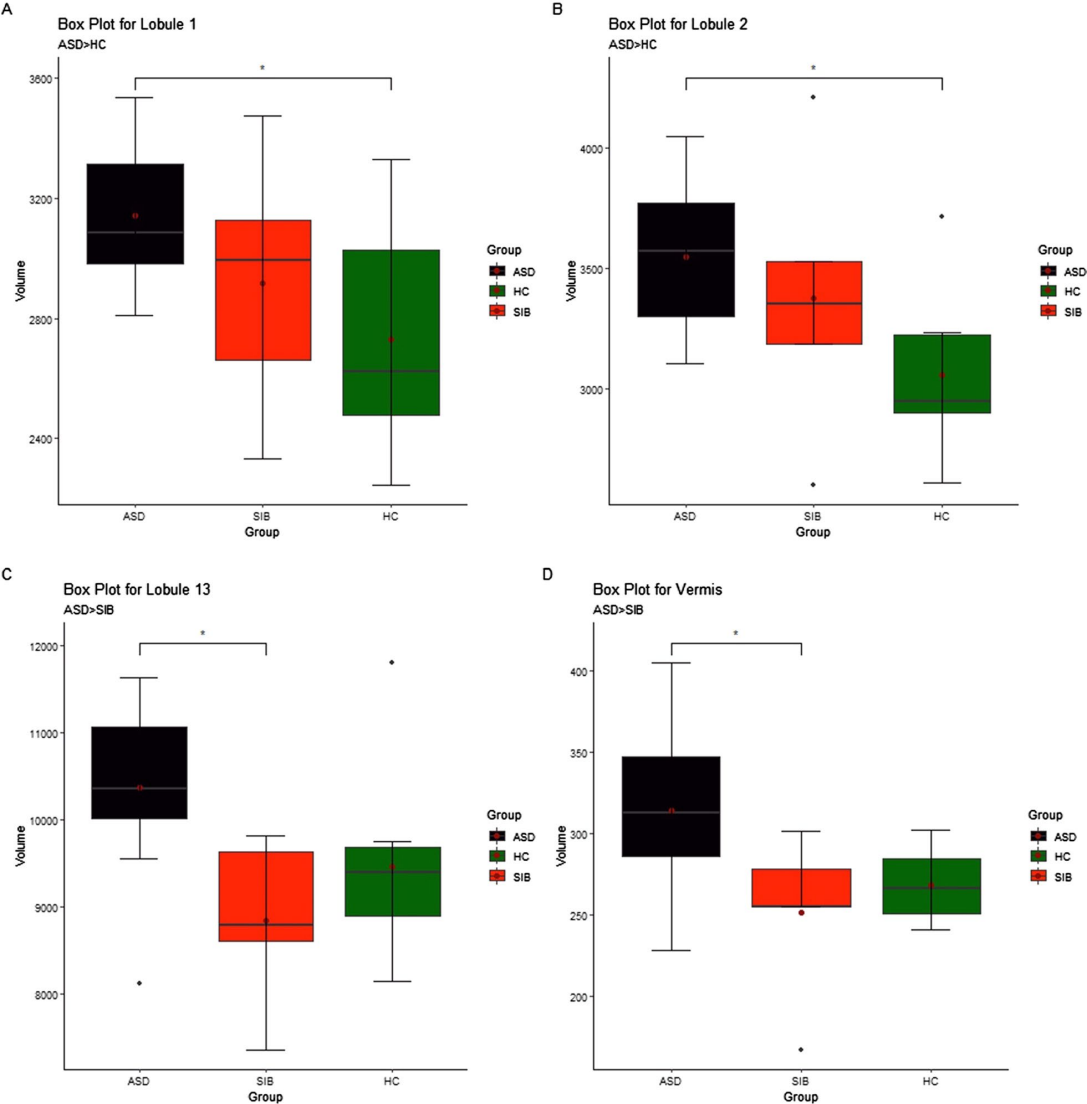
Box-Whisker plots from the representative cerebellar lobules demonstrating the volumetric changes between these three groups, i.e., ASD, ASD siblings, and healthy controls. Asterisk (*) represents the level of statistical significance between these groups obtained using ANOVA analysis. Cerebellar lobular volumes are represented in mm^3^ (represented in the Y-axis), and the X-axis represents the participating groups. *ASD* Autism spectrum disorder, *SIB* ASD sibling, and *HC* Healthy controls, respectively

Similar to our neurodevelopmental data, we have also observed significantly altered cerebellar volume at the lobular levels from the specific lobules among the three groups, i.e., ASD, ASD siblings, and healthy controls, at an early age (Fig. 3). Multiple comparisons using Bonferroni correction, we observed significantly higher cerebellar volume of children with ASD compared to healthy controls in left and right I–IV (Lobule 1 and 2) (Table 2, and Fig. 3a and b). While comparing cerebellar lobular volume between ASD and ASD siblings, we found several cerebellar regions demonstrating a significant increase in lobular volume in the ASD group, including right CrusII (lobule 13), right VIIIa and VIIIb (lobule 16 and 19), and Vermis (lobule 27), respectively (Table 2 and Fig. 3c and d). We also observed significantly higher lobular volumes in Left V (lobule 3) and between children with ASD and healthy control; however, it did not reach statistical significance after multiple comparisons. Similarly, right VIIIb (lobule 22) also demonstrated an increase in lobular volume in the ASD group compared to ASD siblings, but after multiple comparison, no statistical significance was observed (Table 2).

**Table 2:** Comparison of cerebellar lobular volumes between ASD, ASD siblings, and healthy controls

Cerebellar regions/ Lobules	F	p-value	Multiple Comparisons (Bonferroni)	p-value
Left I-IV (Lobule 1)	3.951	0.034	ASD > HC	0.032
Right I-IV (Lobule 2)	4.123	0.03	ASD > HC	0.027
Left V (Lobule 3)	3.728	0.04	No significant in multiple comparisons	NA
Right CrusII (Lobule 13)	4.626	0.021	ASD > ASD-siblings	0.028
Right VIIb (Lobule 16)	4.918	0.017	ASD > ASD-siblings	0.024
Right VIIIa (Lobule 19)	4.469	0.024	ASD > ASD-siblings	0.041
Right VIIIb (Lobule 22)	3.747	0.04	No significant in multiple comparisons	NA
Vermis (Lobule 27)	4.644	0.021	ASD > ASD-siblings	0.042

Along with altered lobular cerebellar volume between these groups, we also observed that multiple lobular regions demonstrate a significant correlation between various neurodevelopmental scores. These cerebellar lobules are specifically implicated with socio-behavioral, visuospatial, motor, and cognitive functions (Table 3). Interestingly, we did not observe any significant difference in total cerebellar volume between these three groups.

A. **Comparison of cerebellar lobular volume:**
  I. **ASD Vs. Healthy controls:** We observed significantly increased cerebellar lobular volume in left and right I-IV (lobules 1 and 2) between ASD and healthy controls after performing multiple comparisons between these groups (Table 2 and Fig. 3a, b).
  II. **ASD vs. ASD siblings:** Interestingly, like ASD to healthy controls, we also observed cerebellar lobular volumetric differences between ASD and ASD siblings in the following lobules right CrusII (lobule 13), Right VIIb (lobule 16) and right VIIIa (lobule 19) and Vermis (Lobule 27); respectively (Table 2 and Fig. 3c, d).
  III. **ASD-sibling vs. Healthy control:** It was intriguing to note that no statistical significance was observed in any lobular volumes between these groups (Table 2, and Fig. 3c).
B. **Correlation between cerebellar lobular volume and developmental measures:**
  1. **Vineland social maturity scale:**
    VSMS measures the social quotient of developing children. The social quotient (SQ) score is calculated by dividing social age (SA) by chronological age (CA) and multiplying by 100. SQ scores of less than 70 indicate significant deficits in an individual's adaptive functioning. SQ scores correlate with standard intelligence measures (IQ), though it is not a direct measure of IQ.
    Our results demonstrate a significant correlation between the SQ derived from the VSMS scores and cerebellar lobular volumes, including left VI (lobule 5) and left and right Crus I and II (lobule 8, 10, 11, and 13), respectively, in children with ASD (Table 3). Nonetheless, no significant correlation was observed between the SQ scores and total cerebellar volume, and also, we could not observed a statistically significant correlation between cerebellar lobular volumes.
    On the other hand, we also observed a correlation between lobule volumes and SQ scores in ASD siblings, including left and right I–IV and V (lobules 1–4). We also watched three additional lobules, including right CrusI (Lobule 10), left CrusII (lobule 11), and right IX (lobule 25), respectively, in the ASD sibling.
    Interestingly, we also observed that out of 34 cerebellar lobular volumes, only two demonstrate statistically significant correlations in healthy controls, including lobule right VIIIb (lobule 22) and vermis (lobule 27), respectively. However, we must note that we observed a negative correlation between lobular volume and SQ scores with healthy controls. In contrast, a positive correlation was noted between lobular lobules and SQ scores in ASD and ASD-sibling groups (Table 3).
  2. **Bayley scales of infant and toddler development:**
    The Bayley scale has been in practice for several decades as a valuable tool for detecting early developmental delay in clinical and research settings. Bayley tools can assess behavioral, motor, and mental domains in developing children between 1 to 42 months of age. In this study, we have used Bayley-III to determine the following occupation of the development in children with ASD, ASD siblings, and healthy controls.
C. **Cognition domain:** Cognition is measured by whether the toddlers can find the hidden object, the presence of object permanence, know shapes and color concepts, representative and relational play, pay attention to the story, solve simple puzzles, etc. There was no statistically significant correlation between composite cognitive scores derived from the Bayley assessment and lobular volumes found in children with ASD or with healthy control. However, we observed a significant negative correlation between cognitive scores and lobular volume (Left VI lob 5) in ASD siblings (Table 3).
D. **Language domain:** Receptive language measures how much the toddler can understand, and expressive language measures how much the toddler can articulate the speech. Response to name calling, imitation, pointing, identifying, and documenting the objects in the environment, and a vocabulary of 250 words by age three are expected in typically developing toddlers. We performed a Pearson correlation to find a correlation between lobular volume changes and language scores derived from the Bayley assessment. There was no correlation between composite language scores with lobular volumes in children with ASD and ASD siblings. On the other hand, we observed a significant positive correlation between language scores and lobular volumes (Left CrusI lob 8) in healthy controls (Table 3).
E. **Motor domain:** The motor domain measures fine and gross motor skills, and precisely the fine motor skills measure the toddler's eye-hand coordination and finger dexterity. Activities that are looked to measure fine motor skills are pincer grasp, spontaneous scribbling, putting coins in the piggy bank, putting beads in the thread, stacking the cubes, etc., and gross skills are sitting without support, independent walking, climbing stairs, balancing the foot while dressing, peddling tricycle, etc. There was no correlation between composite language scores with lobular volumes in children with ASD and ASD siblings. We observed a significant negative correlation between motor scores derived from the Bayley assessment with lobular volume (Left VIIIa Lob 17) in healthy controls (Table 3).

**Table 3:** Pearson correlation between lobular volumes and behavioral scores from ASD, ASD siblings, and healthy controls

		**ASD**		**ASD siblings**		**HC**	
**Behaviour measures**	**Cerebellar lobules**	**r**	**p-value**	**r**	**p-value**	**r**	**p-value**
SQ Scores	Left I-IV (Lobule 1)	–	–	0.99	0.001	–	–
	Right I-IV (Lobule 2)	–	–	0.95	0.011	–	–
	Left V (Lobule 3)	–	–	0.97	0.007	–	–
	Right V (Lobule 4)	–	–	0.92	0.025	–	–
	Left VI (Lobule 5)	0.58	0.048	–	–	–	–
	Left CrusI (Lobule 8)	0.8	0.002	–	–	–	–
	Right CrusI (Lobule 10)	0.83	0.001	0.94	0.016	–	–
	Left CrusII (Lobule 11)	0.64	0.023	0.9	0.037		
	Right CrusII (Lobule 13)	0.58	0.047	–	–	–	–
	Right VIIIb (Lobule 22)	–	–	–	–	−0.97	0.029
	Right IX (Lobule 25)			0.94	0.017		
	Vermis (Lobule 27)	–	–	–	–	−0.98	0.013
**Composite scores of Cognitive, Language, and Motor**							
CC Scores	Left VI (Lobule 5)	–	–	-0.88	0.048	–	–
LC Scores	Left CrusI (Lobule 8)				–	0.99	0.003
MC Scores	Left VIIIa (Lobule 17)	–	–	–	–	−0.97	0.027

*SQ scores* Social quiescent scores, *CC scores* cognitive composite scores, *LC scores* Language composite scores, and *MC scores* Motor composite scores, respectively

## Discussion

In this study, we performed a cerebellar lobular level analysis of MRI data and its correlation with socio-behavioral and developmental data in children between 15 and 36 months of age with ASD, ASD siblings, and healthy controls. In the current study, using structural MRI data, we observed significant differences in the cerebellar lobular volumes among the three groups, with a substantial increase in gray matter volume in specific lobules like the cerebellar vermis and CrusII, and right VIIb, VIIIa, and VIIIb in children with ASD compared to healthy controls and ASD siblings. The altered lobular volumes were also correlated with clinical and behavioral measures like SQ and cognitive, motor, and language scores derived from VSMS and Bayley-III measurements. However, on the other hand, there were no statistically significant differences between these groups when compared to the total cerebellar volume. Similarly, we did not find any statistically significant correlation between total cerebellar volume and behavioral scores. To the best of our knowledge, this is the first study that looked at cerebellar volumetric differences at an early age with three groups, i.e., Children with ASD, ASD siblings, and typically developing children.

Neuroanatomical diversity accounts for a substantial proportion of the risk for ASD [30]. Though several candidates of neuroanatomical biomarkers have been proposed, there is no clarity on the exact neuroanatomical traits more strongly associated with ASD symptomatology. But, in the scientific studies at a younger age, one of the most consistent findings from early studies of brain development in ASD is that head size is normal at birth, but brain size is significantly enlarged by 2–3 years of age [17, 32–34]. Researchers have reported that disruption in the cerebellum in the initial years of life positively correlates with clinical features of autism [6, 13, 17, 35]. The scientific literature has been well documented that damage to the cerebellar hemispheres leads to delays in language, verbal reasoning, and deficits in visual coordination. Impaired vermis volume is associated with social withdrawal, impaired gaze, anxiety, and stereotyped behavior [12, 34]. Persistent, altered cerebellar volumetric differences emerge in the first two years of life. A similar pattern of increased cerebellar volume at an early age was also noted in children with ASD compared to healthy controls [12, 33, 35]. In the current study, our findings are concordant with previously reported altered volumes in various cerebellar regions, including the cerebellar vermis. It has been reported that the primary function of the vermis, which represents the spinocerebellum, is associated with the coordination of motor movements and maintenance of muscular tone. Recent cerebellar studies have also reported that cerebellar lobules (Crus I and Vermis) are also associated with social cognition, and deficits in social cognition are a hallmark of ASD symptoms.

The specific relationship between brain and behavior has been reported in children with ASD, which modulate with temporal specificities like higher growth rates of brain volume between 12 and 24 months (but not between 6 and 12 months) were associated with greater severity scores in the social skill deficits but not in the repetitive behavior domain at 24 months [36]. Interestingly, we also observed significantly increased lobular volume in the vermis in children with ASD (age 12–36 months) compared to healthy controls and ASD siblings (Table 2). This altered lobular volume was also correlated with social and cognitive scores of the behavioral parameters (Table 3). However, there were no significant differences in lobular volume between ASD siblings and healthy controls. Our findings demonstrate that these altered lobular volumetric differences may be due to abnormal neuronal pruning at an early age in children with ASD. Recent studies of early brain and behavior development have provided important new insights into ASD pathophysiological conditions [37]. ASD-specific brain imaging features have been identified as early as six months of age, and age-specific brain and behavior changes have been demonstrated across the first two years of life, highlighting the developmental nature of ASD.

Multiple studies have reported an early change in brain volume in various brain regions, including the cerebellum, in children with ASD [38, 39]. Subsequently, their brain expansion reverse and volume start demonstrating the trend at around five years of age. The most implicated cerebellar structure is the vermis in children with ASD, and our findings are also consistent with previous studies [35, 40–45]. It has been reported that the posterior CrusII of the cerebellum is specialized for social mentalization and emotional self-experiences [46]. On the other hand, the posterior cerebellar lobules, including Crus I, II, and VI, are associated with clinical symptoms of ASD, like language processing skills [47].

Our research helps us to understand the neurobiology of cerebellar development in children with ASD and ASD siblings compared to typically developing children. It is vital because the cerebellum is compensable concerning motor functions. Still, cognitive and social processes are specifically vulnerable in early life due to perturbation of the cerebellar development and indicate the sensitive-period mechanism for brain development. It is the first prospective study from an Indian cohort where the MRI data was collected under natural sleep among toddlers. The cerebellar structure and function are confined to understanding movement disorders such as spinocerebellar ataxia and other neurodegenerative disorders; however, the current study more precisely demonstrates the implications of altered volumetric changes for ASD and other related neurodevelopmental disorders.

Considering the technical challenges and several other study-related difficulties, including performing MRI scans under natural sleep, here we would also like to point out some of the limitations of this study. The sample size included in this study is small; however, considering the unprecedented situations over the last couple of years, for the global pandemic, we struggled to recruit the subject in this study. This is a cross-sectional study; however, as the developmental age of the study population, a longitudinal study would be warranted in the future for a better understanding of the brain-behavior relationship over a period of time, as we know very well that most ASD children with the early behavioral intervention will perform better in the later age of life. Hence, a longitudinal MRI study will provide a better understanding of the brain neuroanatomical and as well as structural changes over a while. Hence, future studies with a larger sample size must confirm the study finding and replicate and translate the finding for a larger population. Due to the smaller sample size and the limited number of gender-specific subjects available, we could not consider gender as a variable factor in our analysis. Future studies with better sample sizes, including a balanced gender ratio, may provide a more specific gender-related effect on brain-behavior functions in children with ASD. However, it is well-documented in the scientific literature that males are four times more susceptible to ASD than females. Hence, it is expected to have fewer female children with ASD compared to male ASD children.

## Conclusions

Our findings demonstrate that early brain imaging is promising for early targeted interventional therapies in children with ASD. There is a general understanding in medicine that earlier treatment has better outcomes, and in ASD, there is an emerging consensus that earlier intervention results in more successful outcomes. These findings open a new perspective for considering the cerebellum as a potential target for implementing early interventional therapies. Examining early brain volumetric and behavior trajectories also has the potential to parse the etiologic heterogeneity in ASD, a well-recognized impediment to developing targeted, mechanistic treatments. Findings from this study highlight the current state of the science in the pursuit of early brain and behavioral markers of ASD during early childhood and examine the potential implications of these findings for treating ASD-like conditions.

## Data Availability

This published article includes all the data generated or analyzed during this study. The reported data is also available with the corresponding author and can be accessed upon submitting a reasonable request.

## Abbreviations

ASD: Autism spectrum disorder
GM: Gray matter
MRI: Magnetic resonance imaging
SUIT: Spatially Unbiased Infratentorial Template
WM: White matter
